# Cohort profile: The Endometriosis pain QUality aftEr Surgical Treatment (EndoQUEST) Study

**DOI:** 10.1371/journal.pone.0269858

**Published:** 2022-06-13

**Authors:** Amy L. Shafrir, Allison F. Vitonis, Britani Wallace, Amy D. DiVasta, Jenny Sadler Gallagher, Naoko Sasamoto, Marc R. Laufer, Kathryn L. Terry, Stacey A. Missmer

**Affiliations:** 1 Division of Adolescent and Young Adult Medicine, Department of Pediatrics, Boston Children’s Hospital and Harvard Medical School, Boston, Massachusetts, United States of America; 2 Boston Center for Endometriosis, Brigham and Women’s Hospital and Boston Children’s Hospital, Boston, Massachusetts, United States of America; 3 Department of Obstetrics and Gynecology, Brigham and Women’s Hospital and Harvard Medical School, Boston, Massachusetts, United States of America; 4 Division of Gynecology, Department of Surgery, Boston Children’s Hospital and Harvard Medical School, Boston, Massachusetts, United States of America; 5 Department of Epidemiology, Harvard T.H. Chan School of Public Health, Boston, Massachusetts, United States of America; 6 Department of Obstetrics, Gynecology and Reproductive Biology, College of Human Medicine, Michigan State University, Grand Rapids, Michigan, United States of America; Dipartimento di Scienze Mediche e Chirugiche (DIMEC), Orsola Hospital, ITALY

## Abstract

Endometriosis affects reproductive-aged females and varies considerably in terms of symptom presentation, morphologic features, and treatment response. Most studies investigating symptom recurrence after an endometriosis-related surgery have been conducted among adults. The Endometriosis pain QUality aftEr Surgical Treatment (EndoQUEST) Study was established to assess characteristics and biomarkers that are associated with pain remediation and improved quality of life after an endometriosis-related surgery among adolescents and young adults. This paper describes the EndoQUEST methodology, summarizes baseline descriptive factors, and compares characteristics by participant retention status. We enrolled 100 surgically-confirmed endometriosis participants aged 12–23 years who provided questionnaire data on reproductive and behavioral factors, pain characteristics and quality of life at three time points; before surgery, 6 weeks to 26 weeks after surgery, and 1 year after surgery. Among these 100 participants, 88 provided blood and/or saliva at all three time points, while 12 provided blood and/or saliva samples only before surgery and 6 to 26 weeks after surgery. There was little evidence of lost to follow-up at 1 year after surgery due to pain symptoms, as pain and quality of life characteristics were similar between participants who completed the questionnaire 1 year after surgery and those who did not. Analyses utilizing these longitudinal data will advance personalized treatment decision making for adolescents and young adults with endometriosis.

## Introduction

Endometriosis, characterized by endometrial-like tissue thriving outside the uterus, affects ~10% of reproductive-aged females, and can lead to debilitating pelvic pain and diminished quality of life (QoL) [1]. While a “gold standard” diagnosis requires surgical visualization of lesions with histologic confirmation, this invasive diagnostic contributes to an average 7-year delay from symptom onset to diagnosis [1]. Although two-thirds of adults report that their endometriosis-associated symptoms started during adolescence [2], endometriosis remains understudied among adolescents and young adults (AYA).

Although AYA are more likely to present with revised American Society of Reproductive Medicine (rASRM) endometriosis stage I/II disease compared with adult women [3, 4], most studies evaluating pain and QoL following endometriosis-related surgery focused on AYA with rASRM stage III/IV disease [5–7]. Of the studies among AYA with stage I/II disease, 24–80% of participants reported complete symptom resolution or a significant improvement after surgery [8–11]. However, the studies mainly utilized medical record review and clinic encounters to assess symptoms post-surgery–potentially leading to bias if patients with unchanged or worsened symptoms were less likely to continue care within the same surgical practice. Further, no published study to date has collected biologic samples pre- and post-endometriosis surgery in an adolescent population presenting with pelvic pain.

To address this knowledge gap, we conducted a longitudinal cohort study of AYA undergoing an endometriosis-related surgery, including the assessment of pain symptoms, QoL, and biomarkers before surgery and at two time points post-surgery. Results of the EndoQUEST study will enable the identification of participant-related factors that distinguish endometriosis patients who will experience pain and QoL improvement after surgery from those who will not to advance personalized medical decision making. This paper provides a profile of the EndoQUEST cohort study design, including pre- and post-surgery factors related to retention through end of follow-up.

## Materials and methods

### Study design

**Fig 1:**
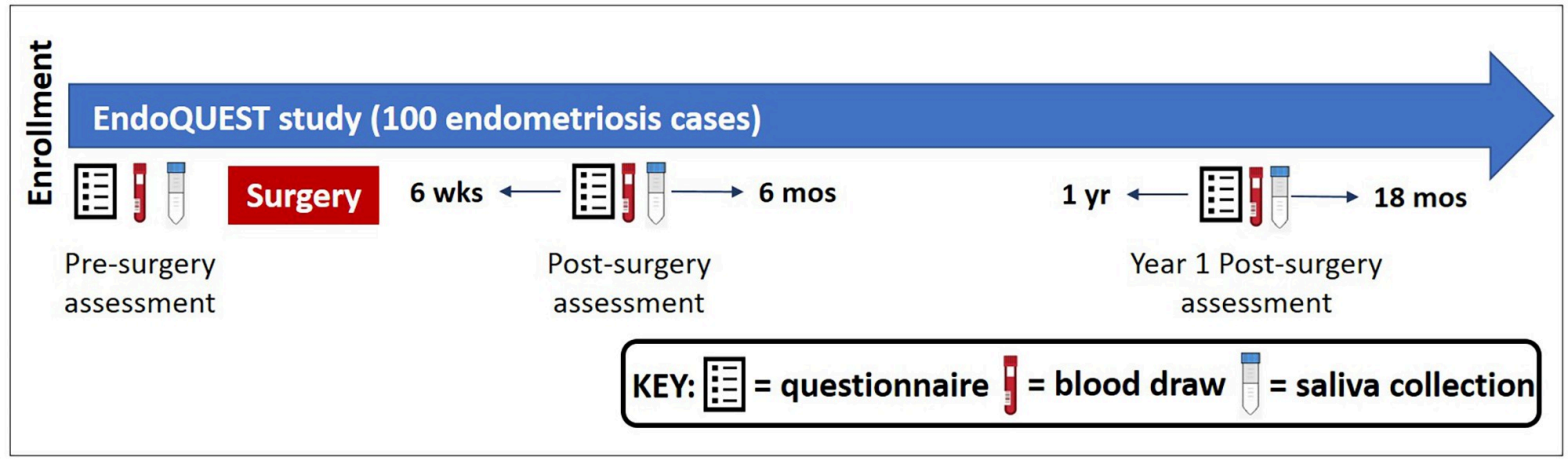
Schematic of the EndoQUEST questionnaire and biospecimen collection.

EndoQUEST is a longitudinal, prospective cohort study. Questionnaire data on pain symptoms, QoL, demographics, reproductive, and medical history as well as blood and saliva samples, were collected: (i) prior to an endometriosis-related surgery (pre-surgery), (ii) between 6 and 26 weeks after surgery (post-surgery), and (iii) one year after surgery (Y1 post-surgery) (Fig 1). The study was approved by the Boston Children’s Hospital (BCH) Institutional Review Board (Assurance Identification #: FWA00002071; IRB Registration #: IRB00000352 and IRB00010042) on behalf of both BCH and Brigham and Women’s Hospital (BWH). Written informed consent was obtained, with parental consent and participant assent for girls <18 years of age at enrollment.

### Participant recruitment

**Fig 2:**
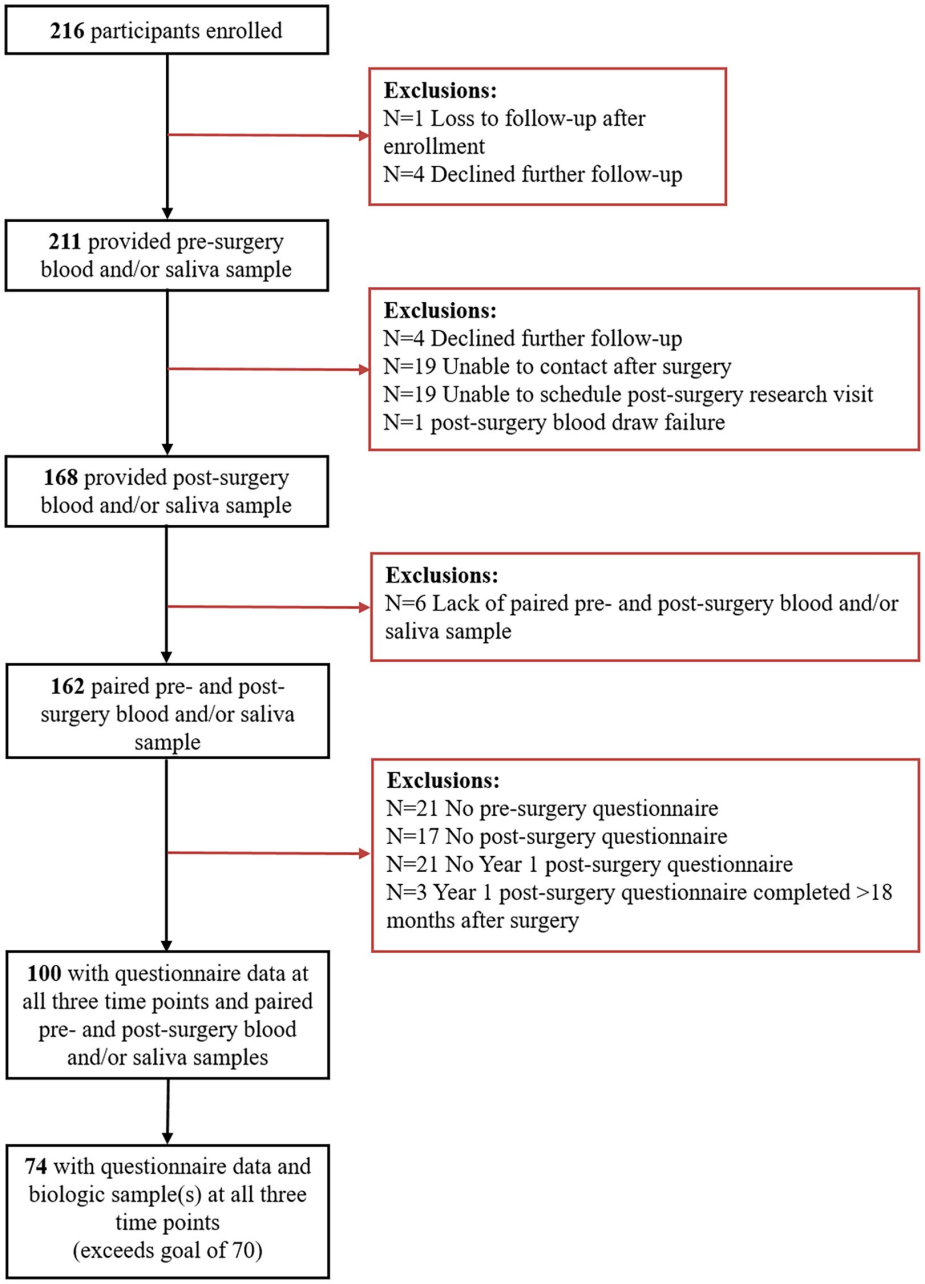
Inclusion (black boxes) and exclusion (orange boxes) criteria for the EndoQUEST analytic sample.

Participants were enrolled between February 2013 and January 2017 from the Women’s Health Study: from Adolescence to Adulthood (A2A) cohort, an ongoing longitudinal cohort oversampled for adolescents and women with endometriosis and described previously [12]. A2A participants with endometriosis were recruited from BCH and BWH clinics. Eligible participants were (i) female, (ii) between the ages of 8 and 25 years, (iii) able to read and understand English, and (iv) scheduled to have an endometriosis-related surgery within the next six months. Participants without endometriosis visualized at their surgery were excluded. In total, 216 participants were enrolled into the EndoQUEST study (Fig 2), with 100 participants having a complete compliment of questionnaire data at all three time points and at least one paired pre- and post-surgery blood or saliva sample.

### Endometriosis surgery

All participants underwent an endometriosis-related laparoscopic surgery with the same surgeon (MRL). Operative data on recent hormone use, surgical indication, rASRM score, lesion locations and colors, and presence of adhesions was collected on a surgical form compliant with the World Endometriosis Research Foundation (WERF) Endometriosis Phenome and Biobanking Harmonization Project (EPHect) recommended forms [13, 14]. Endometriosis was identified using a “close tip” technique. Deep lesions of endometriosis were excised and superficial peritoneal lesions of endometriosis were destroyed utilizing electrocautery. Identified adhesions were lysed.

### Participant questionnaires

Participants completed questionnaires at all three time points utilizing REDCap electronic data capture tools [15]. To increase questionnaire completion, participants who did not complete the pre-surgery questionnaire after three follow-ups were mailed a shorter, paper version of the questionnaire. Participants who did not complete the Y1 post-surgery questionnaire after three follow-ups were mailed a paper copy of the full questionnaire.

### Pre-surgery questionnaire

Early in EndoQUEST enrollment, while actively harmonizing with the WERF EPHect clinical questionnaire, we modified the pre-surgery questionnaire twice (Versions 1 and 2). Seven participants completed version 1 and 11 participants completed version 2. Versions 1 and 2 were very similar but did not include all of the WERF EPHect compliant questions. Missing items included *in utero* and early childhood exposures and polycystic ovarian syndrome symptoms. In January 2014, we implemented an expanded version of the WERF EPHect clinical questionnaire at the pre-surgery time point [14, 16] and 79 participants completed this version (S1 Table lists the variables added to the standard WERF EPHect questionnaire). Some of the pain variables were directly comparable on the three versions of the pre-surgery questionnaire; others were not. We harmonized the pain variables to ensure we could validly utilize as much of the data as possible. Finally, 3 participants completed a short version of the WERF EPHect clinical questionnaire that included the main demographic, lifestyle, and pain variables.

About 20% of participants (n = 19) completed their pre-surgery questionnaire following their surgery, with time since surgery ranging from 1 to 102 days (median = 28 days). To ensure the integrity of these data, we compared demographics, QoL, and pain symptoms from these 19 participants to the 81 participants who completed their pre-surgery questionnaire prior to surgical intervention (S2 Table). We did not observe substantial differences between the two groups, except that on average the participants with late questionnaire completion reported lower mean Short Form (SF)-12 mental health scores (37.9 vs. 44.8; p-value = 0.04) compared to participants who completed the questionnaire before surgery. Differences in recall periods for the SF-12 (previous 4 weeks) and pain symptoms (previous 3–12 months) most likely explain these results.

### Post-surgery questionnaire

The post-surgery questionnaire included the SF-12, 0–10 numeric rating scales for pain, and information on use of analgesics, opioids, hormone medications, and alternative and complementary therapies (S3 Table).

### Y1 post-surgery questionnaire

The Y1 post-surgery questionnaire was an expanded version of the WERF EPHect standard clinical questionnaire and was virtually identical to Version 3 of the pre-surgery questionnaire.

### Blood and saliva collection

We collected blood and saliva samples pre-surgery, post-surgery, and Y1 post-surgery. Participants completed a questionnaire at sample collection to report date of last menstrual period, timing of last foods/beverages consumed, and recent hormone and other medication use. Blood samples were processed into plasma, serum and buffy coats, and stored at ≤-80°C per WERF EPHect fluids standard operating protocols (with the exception that we centrifuged blood samples at 1790g instead of 2500g, and documented this difference per the minimum WERF EPHect protocol [17]). Saliva samples were stored at ≤-80°C per WERF EPHect fluids standard operating protocols (with the exception that we vortexed saliva samples for 10–15 seconds before freezing instead of centrifuging the saliva at 1000g for two minutes, and documented this difference per the minimum WERF EPHect protocol [17]).

### Change in pelvic pain symptoms

To assess change in pain symptoms one year after surgery, we utilized participant responses on pain severity, frequency, and life interference for acyclic pelvic pain and dysmenorrhea to create a derived variable dichotomized to “pelvic pain improved” vs. “pelvic pain did not improve” by one-year post-surgery. Full details on the creation of this derived variable are in S1 Methods. For each of the pain variables, we classified whether the symptom improved, worsened, or stayed the same. If any of the symptoms worsened or stayed the same, we classified the participant as having not improved by Year 1. If any symptoms improved, without any symptoms worsening, then the participant was classified as having improved by Year 1 post-surgery.

### Sample size determination

The original goal of the EndoQUEST study was to recruit enough participants to establish a cohort of 70 AYA with blood and/or saliva samples collected at all three time points and with complete questionnaire data for conducting longitudinal analyses.

### Data analysis

We assessed the baseline demographics of this cohort, and compared demographics, pain characteristics, and post-surgery health status between participants who completed the Y1 post-surgery questionnaire and those who did not using Fisher’s Exact test and T-tests, as appropriate. Statistical analyses were performed using SAS version 9.4 (SAS Institute Inc, Cary, North Carolina).

## Results

### Blood and saliva collection

Of the 100 participants included in EndoQUEST, 74 had blood and saliva collected at all three time points (pre-surgery, post-surgery and Y1 post-surgery) and 14 had blood and saliva collected only at the pre-surgery and post-surgery visits. One participant had only pre-surgery and post-surgery blood collected (no saliva). Ten participants had only saliva collected (6 provided all three samples and 4 provided only pre- and post-surgery samples).

### Demographics and endometriosis-related characteristics

The mean age of participants was 16.8 years (range = 12–23 years; Table 1). The majority of participants were White (94%), non-Hispanic (95%), reported hormone use prior to surgery (92%) and normal weight (66%). The surgery was first/diagnostic for 87% of participants and was a second or subsequent surgery for 13% (Table 2). Almost all of the participants had rASRM stage I/II endometriosis (98%), and all but one participant had superficial peritoneal lesions only.

**Table 1:** Pre-surgery (at enrollment) demographics of EndoQUEST participants.

	All Participants (N = 100)
Age at surgery (years)	
Mean (SD)	16.8 (2.3)
Median (Min-Max)	16.0 (12–23)
Race, N(%)	
White	94 (94.0)
Other/Unknown	6 (6.0)
Ethnicity, N(%)	
Hispanic	5 (5.0)
Non-Hispanic	95 (95.0)
Hormone use before surgery, N(%)^a^	
No	8 (8.0)
Yes	92 (92.0)
Pain medication use before surgery, N(%)^b^	
No	65 (64.0)
Yes	35 (35.0)
Body Mass Index (kg/m^2^), N(%)	
Underweight	0 (0)
Normal weight	66 (66.0)
Overweight	29 (29.0)
Obese	5 (5.0)

^a^Hormone medications included combined estrogen/progestin contraceptives, progestin-only pills, norethindrone acetate, medroxyprogesterone acetate, GnRH agonists, and progesterone-containing intrauterine devices.

^b^Regular pain medication use was defined as ≥1 time/week over a period of ≥3 months. Examples of pain medications included acetaminophen, aspirin, ibuprofen, COX-2 inhibitors, narcotic analgesics, and muscle relaxants.

**Table 2:** Surgical and endometriosis-related characteristics of EndoQUEST participants.

	All Participants (N = 100)
Type of endometriosis surgery, N(%)	
Second or subsequent surgery	13 (13.0)
First/Diagnostic surgery	87 (87.0)
rASRM stage, N(%)	
Stage I/II	98 (98.0)
Stage III/IV	2 (2.0)
Endometriosis surgical sub-phenotype, N(%)	
Superficial peritoneal lesion(s) only	99 (99.0)
Deep lesion(s)	1 (1.0)
Endometrioma	0 (0.0)
Years between symptom onset and diagnosis	
Median (Min-Max)	3.1 (0–9)
Pain symptoms prompted diagnosis, N(%)^a^	
No	1 (1.0)
Yes	96 (99.0)

^a^Participants were asked “What symptoms, if any, prompted you to see a health care provider before your diagnosis with endometriosis?” with the option of pain, infertility, no symptoms, and other. Three participants were missing whether pain symptoms prompted their endometriosis diagnosis

### Comparison of Y1 questionnaire completers and non-completers

Compared to the 100 participants who completed the Y1 post-surgery questionnaire, the 24 non-completers had similar pre-surgery and 6 to 26 week post-surgery demographics and pain symptoms (Table 3). The non-completers were slightly more likely to report that their acyclic pelvic pain interfered with work/school (82% vs. 65%; p = 0.24) and daily activities at home (71% vs. 59%; p = 0.57) compared to Y1 post-surgery questionnaire completers, although severity and frequency of acyclic pelvic pain were similar between the two groups. Participants who did not complete the Y1 post-surgery questionnaire were more likely to report lower SF-12 physical health component scores (43.2 vs. 48.7; p = 0.02) and a greater maximum severity of acyclic pelvic pain and dysmenorrhea (6.4 vs. 5.4 on 0–10 pain rating scale; p = 0.22) on the 6 to 26 week post-surgery questionnaire compared to those who did complete the Y1 post-surgery questionnaire.

**Table 3:** Comparison of pre- and post-surgery characteristics for participants who completed and did not complete the Y1 post-surgery questionnaire.

	Completed Year 1 post-surgery questionnaire	Did not complete Year 1 post-surgery questionnaire	
	N = 100	N = 24	p-value^a^
	**Pre-Surgery Characteristics** ^b^		
Age (years)			
Mean (SD)	16.8 (2.3)	16.4 (2.4)	0.52
Race, N(%)			
White	94 (94.0)	20 (83.3)	0.10
Other/Unknown	6 (6.0)	4 (16.7)	
Ethnicity, N(%)			
Hispanic	95 (95.0)	22 (95.7)	0.99
Non-Hispanic	5 (5.0)	1 (4.3)	
Hormone use, N(%)			
No	8 (8.0)	3 (12.5)	0.44
Yes	92 (92.0)	21 (87.5)	
Pain medication use, N(%)			
No	65 (65.0)	15 (62.5)	0.82
Yes	35 (35.0)	9 (37.5)	
rASRM stage, N(%)			
Stage I/II	98 (98.0)	24 (100)	0.99
Stage III/IV	1 (2.0)	0 (0)	
Endometriosis surgical sub-phenotype, N(%)			
Superficial peritoneal lesion(s) only	99 (99.0)	24 (0)	0.99
Deep infiltrating	1 (1.0)	0 (0)	
Age at symptom onset (years)^c^			
Mean (SD)	13.5 (2.2)	13.1 (2.1)	0.46
Time between symptom onset and diagnosis (years)^c^			
Mean (SD)	3.1 (2.1)	2.8 (2.0)	0.63
Type of endometriosis surgery, N(%)			
Second or subsequent surgery	13 (13.0)	2 (8.3)	0.73
First / Diagnostic surgery	87 (87.0)	22 (91.7)	
SF-12 Mental health component^d^			
Mean (SD)	43.7 (12.2)	41.7 (11.2)	0.49
SF-12 Physical health component^d^			
Mean (SD)	44.7 (10.8)	44.6 (9.9)	0.96
Acyclic pelvic pain in last 3 months, N(%)^e^			
No	35 (35.0)	6 (27.3)	0.47
Yes	65 (65.0)	16 (72.7)	
Severity of acyclic pelvic pain in last 3 months^f^			
Mean (SD)	7.6 (2.1)	7.8 (1.9)	0.70
Frequency of acyclic pelvic pain in last 3 months, N(%)^f^			
<1 day/month	4 (6.9)	2 (11.8)	0.93
Monthly but not weekly	15 (25.9)	4 (23.5)	
Weekly	21 (36.2)	6 (35.3)	
Daily	18 (31.0)	5 (29.4)	
Acyclic pelvic pain interfered with work/school, N(%)^f^			
No	21 (35.0)	3 (17.6)	0.24
Yes	39 (65.0)	14 (82.4)	
Acyclic pelvic pain interfered with daily activities at home, N(%)^f^			
No	24 (40.7)	5 (29.4)	0.57
Yes	35 (59.3)	12 (70.6)	
Reported continuous hormone use in last 3 months, N(%)			
No	73 (73.0)	15 (62.5)	0.32
Yes	27 (27.0)	9 (37.5)	
Severity of period pain in last 12 months, N(%)^g^			
Mean (SD)	8.5 (1.5)	7.9 (2.5)	0.27
Usual frequency of period pain in last 12 months, N(%)^g^			
Occasionally	0 (0)	1 (9.1)	0.11
Often	4 (6.7)	1 (9.1)	
Usually	10 (16.7)	0 (0)	
Always	46 (76.7)	9 (81.8)	
Maximum severity of acyclic pelvic pain and dysmenorrhea^h^			
Mean (SD)	8.3 (1.8)	8.1 (2.2)	0.75
	**Post-surgery characteristics** ^i^		
Hormone use, N(%)			
No	1 (1.0)	2 (8.3)	0.10
Yes	99 (99.0)	22 (91.7)	
Pain medication use, N(%)			
No	32 (32.0)	5 (20.8)	0.33
Yes	68 (68.0)	19 (79.2)	
SF-12 Mental health component^j^			
Mean (SD)	46.9 (11.4)	46.8 (10.7)	0.97
SF-12 Physical health component^j^			
Mean (SD)	48.7 (9.6)	43.2 (12.5)	0.02
Maximum severity of acyclic pelvic pain and dysmenorrhea^k^			
Mean (SD)	5.4 (3.3)	6.4 (3.4)	0.22

^a^P-values calculated using T-tests and Fisher’s Exact tests

^b^Pre-surgery characteristics reported on the pre-surgery questionnaire or recorded at endometriosis-related surgery

^c^Missing data for 3 Year 1 non-completers

^d^Missing data for 3 Year 1 completers and 3 Year 1 non-completers

^e^Missing data for 1 Year 1 non-completer

^f^Restricted to participants who reported acyclic pelvic pain in the last 3 months. Note: Missing data for acyclic pelvic pain severity for 2 Year 1 completers and 1 Year 1 non-completer, missing data for acyclic pelvic pain frequency for 7 Year 1 completers, missing data for acyclic pelvic pain interfering with work/school for 5 Year 1 completers, and missing data for acyclic pelvic pain interfering with daily activities for 6 Year 1 completers

^g^Restricted to 73 Year 1 completers and 15 Year 1 non-completers who did not report continuous hormone use in the last 3 months. Note: Missing data for period pain severity for 2 Year 1 completers and missing data for period pain frequency for 13 Y1 completers and 4 Year 1 non-completers

^h^Calculated as the maximum severity of either acyclic pelvic pain in the last 3 months or period pain in the last 12 months. Includes 98 Year 1 completers and 21 Year 1 non-completers

^i^Obtained from the post-surgery questionnaire completed between 6 and 26 weeks after surgery

^j^Missing data for 5 Year 1 completers and 1 Year 1 non-completers

^k^Calculated as the maximum severity of either acyclic pelvic pain in the last 3 months or period pain in the last 12 months. Includes 99 Year 1 completers and 24 Year 1 non-completers

## Discussion

EndoQUEST is a large study of changes in pain symptoms and QoL after an endometriosis-related surgery among AYA with stage I/II endometriosis including detailed questionnaire data and biologic samples. We surpassed our enrollment goal with 74 participants completing questionnaires and providing biologic samples at all three time points. Participants who did and did not respond to the Y1 post-surgery questionnaire were relatively similar in terms of pain symptoms and QoL. This result suggests that response to the Y1 post-surgery questionnaire was unlikely to be related to pain symptoms and QoL before and after surgery–minimizing potential bias due to loss to follow-up.

The study has some limitations. Of the 100 participants, 19 completed their “pre-surgery” questionnaire after surgery. However, no substantial differences were noted between participants who completed the questionnaire at the appropriate time vs. after surgery. To account for potential bias introduced by these 19 participants, we plan to conduct sensitivity analyses excluding these participants. Additionally, as the post-surgery visit was often tied to follow-up visits with the attending gynecologist, there was a wide window within which the post-surgery questionnaire and biospecimen samples were collected (6 to 26 weeks). To investigate the influence of this broad time period, we plan to conduct subgroup analyses restricting to those within smaller portions of the window, e.g., 6 to 13 weeks and >13 to 26 weeks. Further, three versions of the pre-surgery questionnaire were utilized as the questionnaire was refined over the early years of the study. We have harmonized the data across the three versions to validly capture data that can be interpreted consistently. Finally, most EndoQUEST participants are non-Hispanic White females; therefore, the results may not be generalizable to the broader endometriosis population. While the EndoQUEST participants were enrolled from a larger cohort that successfully enrolled 85% of eligible endometriosis patients, there is a need for future research on endometriosis among a more diverse population.

Endometriosis diagnosed in AYA has been significantly understudied and analyses within the EndoQUEST study will assess patient characteristics and biologic markers in relation to pain symptoms at all three time points and to improvement by year 1. The long-term goals of the study are to discover pre-surgery markers of post-surgical pain response and QoL to advance discovery of personalized treatment algorithms.

## Supporting information

S1 Table Additional questions asked on the expanded version of the WERF EPHect clinical questionnaire utilized in the EndoQUEST study. These questions are not included in the standard WERF EPHect clinical questionnaire.
(DOCX)

S2 Table Comparison of participants who completed pre-surgery questionnaire AFTER surgery and participants who completed pre-surgery questionnaire BEFORE surgery. (DOCX)

S3 Table Description of post-surgery questionnaire. (DOCX)

S1 Methods Changes in pain symptoms from pre-surgery to Y1 post-surgery. (DOCX)
