# Supplementary material for: Cohort profile: The Endometriosis pain QUality aftEr Surgical Treatment (EndoQUEST) Study
Source: PLoS One. 2022 Jun 13;17(6):e0269858. doi: 10.1371/journal.pone.0269858 (PMC9191708; doi:10.1371/journal.pone.0269858)
Supplement: S1 Table — These questions are not included in the standard WERF EPHect clinical questionnaire. (DOCX) [file pone.0269858.s001.docx]

**S1 Table.** Additional questions asked on the expanded version of the WERF EPHect clinical questionnaire utilized in the EndoQUEST study. These questions are not included in the standard WERF EPHect clinical questionnaire.

| **Category** | **Variables** |
| --- | --- |
| Demographics | - Birth place - Location lived during most of childhood/adolescence - Current work status - Current relationship status |
| Reproductive/Menstrual characteristics | - Need for medication to have first period - Last time had a natural period - Method of taking birth control pills (regular and get period, regular but continue to take active pills, extended cycle) - Side effects of hormonal medications - Number of months it took to get pregnant - Length of each pregnancy - Infant weight and sex for each pregnancy - Changes in menstrual cycles after birth of last child and after stopped breastfeeding |
| Dysmenorrhea | - Ever seen a doctor for period pain - Age first saw doctor for period pain - Type of doctor first seen for period pain - Number of doctors seen for period pain - Urinary habits when had period pain in the last 3 months |
| Acyclic Pelvic Pain | - Urinary habits when had acyclic pelvic pain in last 3 months - Nausea with acyclic pelvic pain (when taking medications, when eating, during period) - Vomiting with acyclic pelvic pain (when taking medications, when eating, during period) |
| General Pain | - Other types of pain experienced in last 12 months (migraines, pain when bladder is full, abdominal pain, constant burning vaginal pain) |
| Quality of life | - Short Form-36 (pre-surgery) and Short Form-12 (Y1 post-surgery) |
| Polycystic ovarian syndrome characteristics | - Currently have acne - Ever had permanent hair removal from face, chest, or abdomen - Regularly shave, wax, etc. for excess hair on face, chest or abdomen - Rate amount of facial and body hair on upper lip, chin, chest, upper abdomen, lower abdomen, thighs |
| Endometriosis | - Number of previous surgical procedures had for endometriosis or pelvic pain - Age when first saw doctor for endometriosis symptoms - Specialty of doctor first saw for endometriosis symptoms - Number of doctors seen before getting endometriosis diagnosis |
| Family history | - Number of siblings - Family history of breast cancer, ovarian cancer, uterine cancer, menopause before age 46, infertility, two or more miscarriages, polycystic ovarian syndrome - Family history for non-female related conditions - Mother ever mentioned pelvic pain, dysmenorrhea, or endometriosis |
| Early life factors | - Participant birth weight - Participant’s length of gestation - Mother’s birth date - If participant was breastfed as an infant - Exposure to prenatal smoking - Mother used DES while pregnant with participant - Place mother lived the most while pregnant with participant |
| Physical activity | - Does physical activity change by season and activities over the past year by season - Hours per week spent standing/walking or sitting under different circumstances (e.g. school, work, etc.) - Avoid exercise during your period before age 20 |
| Drinking and smoking | - Binge drinking - Current secondhand smoke exposure - Secondhand smoke exposure in childhood |
| Sexual orientation | - Sexual orientation - Sexual partners |
| Sleep | - Amount of sleep each night - Enough sleep each night - Snoring - Sleep interference with day |
| Skin | - Color of natural skin tone before tanning - Amount of moles on body - Number of times in past 5 years had skin checked by doctor or yourself/spouse/partner/parent - Ever had mole cut out by dermatologist or surgeon - Sun exposure and burning - Freckles - Amount of time outdoors in sun at different ages - Number of times sunburned badly that had blisters or soreness lasted for 2+ days or skin peeled - Sunscreen use - Protection measures taken from sun - Tanning bed use |
