# Supplementary material for: Cohort profile: The Endometriosis pain QUality aftEr Surgical Treatment (EndoQUEST) Study
Source: PLoS One. 2022 Jun 13;17(6):e0269858. doi: 10.1371/journal.pone.0269858 (PMC9191708; doi:10.1371/journal.pone.0269858)
Supplement: S2 Table — (DOCX) [file pone.0269858.s002.docx]

**S2 Table.** Comparison of participants who completed pre-surgery questionnaire AFTER surgery and participants who completed pre-surgery questionnaire BEFORE surgery

|  | **Completed pre-surgery questionnaire AFTER surgery^1^** | **Completed pre-surgery questionnaire BEFORE surgery** |  |
| --- | --- | --- | --- |
|  | **N=19** | **N=81** | **p-value**^2^ |
| Age (years) |  |  |  |
| Mean (SD) | 16.7 (2.1) | 16.8 (2.4) | 0.96 |
| Race, N(%) |  |  |  |
| White | 18 (94.7) | 76 (93.8) | 0.99 |
| Other/Unknown | 1 (5.3) | 5 (6.2) |  |
| Ethnicity, N(%) |  |  |  |
| Hispanic | 18 (94.7) | 77 (95.1) | 0.99 |
| Non-Hispanic | 1 (5.3) | 4 (4.9) |  |
| Hormone use, N(%) |  |  |  |
| No | 1 (5.3) | 7 (8.6) | 0.99 |
| Yes | 18 (94.7) | 74 (91.4) |  |
| Pain medication use, N(%) | |  |  |
| No | 13 (68.4) | 52 (64.2) | 0.80 |
| Yes | 6 (31.6) | 29 (35.8) |  |
| rASRM stage, N(%) |  |  |  |
| Stage I/II | 19 (100) | 79 (97.5) | 0.99 |
| Stage III/IV | 0 (0.0) | 2 (2.5) |  |
| Endometriosis subtype, N(%) | |  |  |
| Superficial peritoneal only | 19 (100) | 80 (98.8) | 0.99 |
| Deep infiltrating | 0 (0.0) | 1 (1.2) |  |
| Age at symptom onset (years) |  |  |  |
| Mean (SD) | 13.3 (1.7) | 13.6 (2.3) | 0.55 |
| Time between symptom onset and diagnosis (years) | |  |  |
| Mean (SD) | 2.9 (1.9) | 3.1 (2.1) | 0.72 |
| Type of surgery, N(%) |  |  |  |
| Subsequent | 1 (5.3) | 12 (14.8) | 0.45 |
| Diagnostic | 18 (94.7) | 69 (85.2) |  |
| SF-12 Mental health component^3^ | |  |  |
| Mean (SD) | 37.9 (10.9) | 44.8 (12.1) | 0.04 |
| SF-12 Physical health component^3^ | |  |  |
| Mean (SD) | 44.8 (8.8) | 44.7 (11.2) | 0.98 |
| Acyclic pelvic pain in last 3 months, N(%) | |  |  |
| No | 6 (31.6) | 29 (35.8) | 0.80 |
| Yes | 13 (68.4) | 52 (64.2) |  |
| Severity of acyclic pelvic pain in last 3 months^4^ | |  |  |
| Mean (SD) | 8.0 (2.1) | 7.5 (2.2) | 0.46 |
| Frequency of acyclic pelvic pain in last 3 months, N(%)^4^ | | |  |
| <1 day/month | 0 (0.0) | 4 (8.3) | 0.61 |
| Monthly but not weekly | 2 (20.0) | 13 (27.1) |  |
| Weekly | 3 (30.0) | 18 (37.5) |  |
| Daily | 5 (50.0) | 13 (27.1) |  |
| Acyclic pelvic pain interfered with work/school, N(%)^4^ | |  |  |
| No | 3 (30.0) | 18 (36.0) | 0.99 |
| Yes | 7 (70.0) | 32 (64.0) |  |
| Acyclic pelvic pain interfered with daily activities at home, N(%)^4^ | | |  |
| No | 3 (30.0) | 21 (42.9) | 0.51 |
| Yes | 7 (70.0) | 28 (57.1) |  |
| Reported continuous hormone use in last 3 months, N(%) | | |  |
| No | 13 (68.4) | 60 (74.1) | 0.77 |
| Yes | 6 (31.6) | 21 (25.9) |  |
| Severity of period pain in last 12 months, N(%)^4^ | |  |  |
| Mean (SD) | 8.5 (1.4) | 8.5 (1.5) | 0.96 |
| Usual frequency of period pain in last 12 months, N(%)^5^ | | |  |
| Often | 0 (0.0) | 4 (8.5) | 0.86 |
| Usually | 2 (15.4) | 8 (17.0) |  |
| Always | 11 (84.6) | 35 (74.5) |  |
| Maximum severity of acyclic and dysmenorrhea^6^ | |  |  |
| Mean (SD) | 8.4 (1.6) | 8.3 (1.8) | 0.79 |

1. 16 completed the pre-surgery questionnaire within 60 days after their surgery and 3 completed the questionnaire within 102 days.

2. T-test and Fisher’s Exact tests were used to calculate p-values

3. Missing data for SF-12 Mental and Physical health component for 3 participants who completed pre-surgery questionnaire after surgery

4. Restricted to participants who reported acyclic pelvic pain in the last 3 months Note: Missing data for acyclic pelvic pain severity for 1 participant who completed pre-surgery questionnaire after surgery and 1 participants who completed pre-surgery questionnaire before surgery, missing data for acyclic pelvic pain frequency for 3 participants who completed pre-surgery questionnaire after surgery and 4 participants who completed pre-surgery questionnaire before surgery, missing data for acyclic pelvic pain interfering with work/school and interfering with daily activities for 3 participants who completed pre-surgery questionnaire after surgery and 2 participants who completed pre-surgery questionnaire before surgery

5. Restricted to participants who did not report continuous hormone use in the last 3 months and completed Version 3 of pre-surgery questionnaire. Note: 2 participants who completed the pre-surgery questionnaire before surgery reported “No” menstrual pain and are not included in the severity or frequency variables; 11 participants completed Versions 1 and 2 of the pre-surgery questionnaire and are not included in this variable

6. Calculated as the maximum severity of either acyclic pelvic pain in the last 3 months or period pain in the last 12 months. Includes 18 participants who completed pre-surgery questionnaire after surgery and 80 participants who completed pre-surgery questionnaire before surgery
