# Supplementary material for: Cohort profile: The Endometriosis pain QUality aftEr Surgical Treatment (EndoQUEST) Study
Source: PLoS One. 2022 Jun 13;17(6):e0269858. doi: 10.1371/journal.pone.0269858 (PMC9191708; doi:10.1371/journal.pone.0269858)
Supplement: S3 Table — (DOCX) [file pone.0269858.s003.docx]

**S3 Table.** Description of post-surgery questionnaire

| **Measure** | **Description** |
| --- | --- |
| Pain rating | 0-10 pain scale (0=no pain, 10=worst pain imaginable) for today, best day this week, worst day this week, best day this month, worst day this month |
| Current medication use | Over the counter pain medications (naproxen, aspirin, ibuprofen, acetaminophen) and prescription pain medications (diclofenac, Naprosyn, other) – instructed to check all that apply |
| Current hormone therapy use | Birth control pill, injectable progestin, hormone ring, norethindrone acetate, progestin-only pill, implant, hormone patch, Lupron depot injections – instructed to check all that apply |
| Other medication use | Space to write in names of other medications currently taking |
| Current medication side effects | Headache, nausea, vomiting, uterine bleeding, fatigue, dizziness, hot flashes, constipation, mood changes, weight gain, muscle pain, joint pain, other – instructed to check all that apply |
| Complementary and alternative therapies used | Meditation, music, massage, ice, heating pad, hot bath, TENS unit, yoga, other physical activity, acupuncture, sleep, other – instructed to check all that apply |
| Menstrual period timing | Asked to record date of last menstrual period or indicate that the participants was not having menstrual periods |
| Short Form (SF)-12 | 12 questions to assess physical and emotional health with scores ranging from 0 to 100 (0=worst physical and emotional functioning, 100=best physical and emotional functioning). Divided into subscales: physical health component (0-100) and mental health component (0-100). |
