# Supplementary material for: Cohort profile: The Endometriosis pain QUality aftEr Surgical Treatment (EndoQUEST) Study
Source: PLoS One. 2022 Jun 13;17(6):e0269858. doi: 10.1371/journal.pone.0269858 (PMC9191708; doi:10.1371/journal.pone.0269858)
Supplement: S1 Methods — (DOCX) [file pone.0269858.s004.docx]

**S4 Supplemental Methods: Changes in pain symptoms from pre-surgery to Y1 post-surgery**

We did not wish to conflate pain reports with perceived change in symptoms that could erroneously be attributed to specific exposures. Therefore, our modified WERF EPHect compliant questionnaire did not directly ask participants if they had an improvement, worsening or persistence of their endometriosis-related pain symptoms. To quantify change among time periods, we used a three step process to create a derived variable of “Improved by Year 1” and “Did not improve by Year 1”.

**Step 1:**

Changes in pain symptoms from pre-surgery to Y1 post-surgery were calculated for five acyclic pelvic pain variables and six dysmenorrhea variables. Table 1 displays the variables utilized to capture changes in acyclic pelvic pain and the summary classification given to each type of change. The changes were then classified as (1) never had pain (white), (2) symptoms improved (blue), (3) symptoms worsened (orange), and (4) symptoms persisted (grey). Table 2 displays the variables utilized to capture changes in dysmenorrhea and the summary classification given to each type of change.

**Table 1.** Changes from pre-surgery to Y1 post-surgery for acyclic pelvic pain

| **Pre-surgery and Y1 post-surgery questions** | **Pre-surgery and Y1 post-surgery response categories** | **Categories for change between pre-surgery and Y1 post-surgery responses** | **Summary category** |
| --- | --- | --- | --- |
| Any acyclic pelvic pain in the past 3 months | No  Yes | Never had pain | Never had pain |
|  |  | Pain stopped | Symptoms improved |
|  |  | Developed new pain | Symptoms worsened |
|  |  | Pain persisted | Symptoms persisted |
|  |  |  |  |
| Severity of acyclic pelvic pain in past 3 months | 0-10 (0=no pain, 10=worst pain imaginable) | Never had pain | Never had pain |
|  |  | Pain stopped | Symptoms improved |
|  |  | Pain improved by >2 points |  |
|  |  | Developed new pain | Symptoms worsened |
|  |  | Pain worsened by >2 points |  |
|  |  | Pain stayed about the same | Symptoms persisted |
|  |  |  |  |
| Frequency of acyclic pelvic pain in past 3 months | No pain  <1 day per month  Monthly but not weekly  Weekly  Daily | Never had pain | Never had pain |
|  |  | Pain stopped | Symptoms improved |
|  |  | Frequency improved by >1 category |  |
|  |  | Developed new pain | Symptoms worsened |
|  |  | Frequency worsened by >1 category |  |
|  |  | Frequency stayed about the same | Symptoms persisted |
|  |  |  |  |
| Acyclic pelvic pain interfered with work/school | No pain in past 3 months  No  Yes | Never had pain | Never had pain/pain never interfered |
|  |  | Pain never interfered |  |
|  |  | Developed new pain but did not interfere |  |
|  |  | Pain stopped interfering | Symptoms improved |
|  |  | Pain stopped |  |
|  |  | Developed new pain and did interfere | Symptoms worsened |
|  |  | Pain began to interfere |  |
|  |  | Pain continued to interfere | Symptoms persisted |
|  |  |  |  |
| Acyclic pelvic pain interfered with daily activities at home | No pain in past 3 months  No  Yes | Never had pain | Never had pain/pain never interfered |
|  |  | Pain never interfered |  |
|  |  | Developed new pain but did not interfere |  |
|  |  | Pain stopped interfering | Symptoms improved |
|  |  | Pain stopped |  |
|  |  | Developed new pain and did interfere | Symptoms worsened |
|  |  | Pain began to interfere |  |
|  |  | Pain continued to interfere | Symptoms persisted |

**Table 2.** Changes from pre-surgery to Y1 post-surgery for dysmenorrhea

| **Pre-surgery and Y1 post-surgery questions** | **Pre-surgery and Y1 post-surgery response categories** | **Categories for change between pre-surgery and Y1 post-surgery responses** | **Summary category** |
| --- | --- | --- | --- |
| Severity of period pain | No pain  Mild pain  Moderate pain  Severe pain  Reported no periods | Never had pain or never had periods | Never had pain |
|  |  | Pain stopped or periods stopped | Symptoms improved |
|  |  | Pain improved by >1 category |  |
|  |  | Pain started | Symptoms worsened |
|  |  | Pain worsened by >1 category |  |
|  |  | Pain stayed about the same | Symptoms persisted |
|  |  |  |  |
| Severity of period pain in past 12 months | 0-10 (0=no pain, 10=worst pain imaginable) | Never had pain | Never had pain |
|  |  | Pain improved by >2 points | Symptoms improved |
|  |  | Pain worsened by >2 points | Symptoms worsened |
|  |  | Pain stayed about the same | Symptoms persisted |
|  |  |  |  |
| Severity of period pain in past 3 months | 0-10 (0=no pain, 10=worst pain imaginable) | Never had pain | Never had pain |
|  |  | Pain improved by >2 points | Symptoms improved |
|  |  | Pain worsened by >2 points | Symptoms worsened |
|  |  | Pain stayed about the same | Symptoms persisted |
|  |  |  |  |
| Frequency of period pain in past 12 months | Never  Occasionally  Often  Usually  Always | Never had pain | Never had pain |
|  |  | Pain stopped | Symptoms improved |
|  |  | Frequency decreased by >1 category |  |
|  |  | Frequency increased by >1 category | Symptoms worsened |
|  |  | Pain stayed about the same | Symptoms persisted |
|  |  |  |  |
| Had to lie down during last period due to pain | No periods  No  Yes | Never had pain | Never had pain/Did not have to lie down |
|  |  | Never had to lie down |  |
|  |  | Developed new pain but did not have to lie down |  |
|  |  | Pain stopped | Symptoms improved |
|  |  | Had to lie down at pre-surgery only |  |
|  |  | Developed new pain and did have to lie down | Symptoms worsened |
|  |  | Did not have to lie down at pre-surgery but have to lie down at Y1 post-surgery |  |
|  |  | Had to lie down at both time points | Symptoms persisted |
|  |  |  |  |
| Pain prevented going to work or school or carrying out daily activities | No periods  No  Yes | Never had pain | Never had pain/Did not prevent activities |
|  |  | Never prevented activities |  |
|  |  | Developed new pain but did not prevent activities |  |
|  |  | Pain stopped | Symptoms improved |
|  |  | Prevented activities at pre-surgery only |  |
|  |  | Developed new pain and prevented activities | Symptoms worsened |
|  |  | Did not prevent activities at pre-surgery but prevented activities at Y1 post-surgery |  |
|  |  | Prevented activities at both time points | Symptoms persisted |

**Step 2:**

Three dichotomous variables were created to classify each participant as having any/none acyclic pain symptoms that improved, any/none that worsened, and any/none that stayed the same. Then these dichotomous variables were cross-classified to determine if the participant’s acyclic pelvic pain symptoms had improved, worsened, or persisted.

Table 3 displays how the different cross-classification results for the dichotomous variables were combined into (1) symptoms improved, (2) symptoms worsened and (3) symptoms persisted. The same method was used to classify each participant’s overall improvement, worsening, or persistence of dysmenorrhea symptoms.

**Table 3.** Overall classification of endometriosis-related symptom improvement, worsening, or persistence applied separately for acyclic pain and dysmenorrhea

| Symptoms improved | Symptoms improved across all variables |
| --- | --- |
|  | Some symptoms improved and other symptoms persisted |
| Symptoms worsened | Symptoms worsened across all variables |
|  | Some symptoms worsened and other symptoms persisted |
| Symptoms persisted | Some symptoms worsened, other symptoms improved, and other symptoms persisted |
|  | Symptoms persisted across all variables |

**Step 3:**

The overall symptom classification for acyclic pelvic pain and dysmenorrhea was then cross-classified. Table 4 shows all of the cross-classification options, with those colored blue indicating an improvement at year 1 and those colored in orange indicating a worsening or persistence of symptoms at year 1. All of the participants had either dysmenorrhea or acyclic pelvic pain prior to their surgery and thus there were no participants categorized as never having pain for both pain types. This cross-classification was the final step applied for classifying participants as “Improved at Year 1” and “Did not improve at Year 1”.

**Table 4.** Cross-classification of overall symptom change for acyclic pain and dysmenorrhea with categorization of responses into Improved at Year 1 (blue) and Did not improve at Year 1 (orange).

|  | **Acyclic Pelvic Pain** | | | |
| --- | --- | --- | --- | --- |
| **Dysmenorrhea** | **Never had pain** | **Symptoms improved** | **Symptoms worsened** | **Symptoms persisted** |
| **Never had pain** |  | Improved at Y1 | Did not improve at Year 1 | Did not improve at Year 1 |
| **Symptoms improved** | Improved at Y1 | Improved at Y1 | Did not improve at Year 1 | Did not improve at Year 1 |
| **Symptoms worsened** | Did not improve at Year 1 | Did not improve at Year 1 | Did not improve at Year 1 | Did not improve at Year 1 |
| **Symptoms persisted** | Did not improve at Year 1 | Did not improve at Year 1 | Did not improve at Year 1 | Did not improve at Year 1 |
